# Aesthetic Surgery Complications Disproportionately Burden Economically Disadvantaged Patients and Financially Strain Public Health Insurance: A Single Academic Center Retrospective Analysis

**DOI:** 10.1093/asjof/ojae007.055

**Published:** 2024-04-12

**Authors:** Scott Levin, Joseph Firriolo, Granger Wong

**Affiliations:** University of California Davis Medical Center, Sacramento, CA; University of California Davis Medical Center, Sacramento, CA; University of California Davis Medical Center, Sacramento, CA

## Abstract

**Goals/Purpose:**

Complications of elective aesthetic surgery performed in private practice pose significant morbidity to patients and resource burden to healthcare systems. We aimed to characterize contemporary demographic and complication patterns among patients presenting to an academic center after aesthetic surgery, as well as the financial and resource burden of their postoperative care on the hospital and payor.

**Methods/Technique:**

We performed a retrospective review of patients who presented to an urban, academic hospital emergency department in Northern California from September 1, 2020 to September 30, 2023. We included patients with primary complaints related to aesthetic procedures performed at outside centers resulting in a plastic surgery consultation. Demographics, outcomes, hospital charges, and insurance payments were determined. Univariable analyses identified associations with outcomes.

**Results/Complications:**

We identified 36 patients with aesthetic procedure-related complaints. Mean age was 37.5 ± 9.8 years and all patients were female. Compared with the rest of the patient population presenting to the emergency department over the same time period (n=269,286), the identified patients were more likely to be black (34.3% vs. 20.4%, p=.04), Hispanic (40% vs. 24.6%, p=.03), and on Medicaid (80.6% vs. 31.7%, p<.001). Tobacco/cannabis use and obesity were prevalent in 25% and 47.2% of patients, respectively. Most patients underwent aesthetic procedures in the United States (51.4%), followed by Mexico (37.1%) and the Dominican Republic (11.4%). Body regions intervened upon were the abdomen (52.8%), breasts (52.8%), buttocks (33.3%), and arms (8.3%). In 36.1% of patients, multiple body regions were intervened upon during the index case. Abdominal cases included primary abdominoplasty (84.2%), panniculectomy (10.5%), and revision abdominoplasty (5.3%). Breast cases were primary augmentation (36.8%), revision augmentation (21.1%), augmentation mastopexy (15.8%), mastopexy only (15.8%), and other (10.5%). Buttocks cases were gluteal fat grafting (63.6%) and implant insertion (36.4%). Arm cases were all brachioplasty. Median postoperative day was 21 (Interquartile Range [IQR] 11.5, 36). Reasons for presentation included infection (44.5%), dehiscence (16.7%), pain (16.7%), seroma/drainage (11.1%), drain management (5.6%), and hematoma (2.8%). Nearly half received computed tomographic imaging (47.2%). Half of patients were admitted with a median length of stay of 2.5 days (IQR 1, 3). One-third underwent intervention, including implant removal (58.3%), image-guided aspiration (25%), and incision and drainage (16.7%). Patients using tobacco/cannabis were more likely to present with infection (88.9% vs. 25.9%, p=.001) and undergo intervention (66.7% vs. 22.2%, p=.01). There were recurrent emergency department visits among 22.2%. Overall, 44.4% of patients had outpatient follow-up visits for a median of 3 (IQR 2, 4) visits up to a median of 38 (IQR 21.5, 70.5) days after consultation. For the index hospital encounter and any subsequent outpatient/emergency visits, the total median hospital charge was $43,324.96 (IQR $10,728.12, $80,803.18) and median insurance payment was $3,947 (IQR $404.61, $24,516.00). In the setting of operative intervention, median hospital charge and insurance payments were $125,358.70 (IQR $51,065.52, $152,704.70) and $14,863.52 (IQR $3,947, $49,031.13), respectively.

**Conclusion:**

In this case series, the majority of patients presenting with complaints related to aesthetic surgery were economically disadvantaged and nearly half received surgery abroad. Their surgical complications often directly and significantly cost the state-funded health insurance and strained inpatient and outpatient resources. Nearly a quarter of patients were offered aesthetic surgery despite active smoking, and they were more likely to develop infection and receive invasive treatments. While future analysis should aim to replicate our findings in a larger sample, providers and government agencies should warn their vulnerable patients of these risks.

